# Complete mitochondrial genome of the cockle *Anadara antiquata* (Linnaeus, 1758)

**DOI:** 10.1080/23802359.2019.1627931

**Published:** 2019-07-11

**Authors:** Liyun Pu, Hongtao Liu, Guofu Wang, Bingshun Li, Guangyuan Xia, Minghui Shen, Mingqiu Yang

**Affiliations:** Hainan Provincial Key Laboratory of Tropical Maricultural Technologies, Hainan Academy of Ocean and Fisheries Sciences, Haikou, China

**Keywords:** Mitochondrial genome, *Anadara antiquata*, phylogenetic analysis

## Abstract

The complete mitochondrial genome of* Anadara antiquata* was first determined. With a length of 45,454 bp, it consists of 29 tRNA, 2 rRNA, and 17 protein-coding genes (PCGs). The non-coding region was large and atypical around the genome with total 24,162 bp long. The nucleotide composition is significantly biased with AT contents of 62.2%. PCGs have five types of start codon, and terminate with a complete stop codon TAA or TAG. 19 microsatellites (SSRs) were identified in mitogenome sequences. Phylogenetic analysis demonstrated that *A. antiquata* was first clustered with *Anadara vellicate*, then together with *Tegillarca granosa*.

*Anadara antiquata*, commonly known the blood cockle, is an important edible bivalve mollusc which belongs to the family Arcidae, subfamily Anadarinae. It is a commercially valuable fishery species and widely distributed around the Indo-West Pacific region. Recently, it is endangered because the density of its population has decreased rapidly due to high exploitation by humans (Siahainenia et al. 2018). Till now several studies on the biology of *A. antiquata* were reported, such as the fecundity and population (Nurdin et al. 2010; Toral-Barza and Gomez 2012), reproductive cycle and patterns (Toral-Barza and Gomez 2012; Jahangir et al. 2014), hermaphroditism (Afiati 2007), gonad maturation (Afiati 2013), diet (Lim 1966; Kasigwa and Mahika 1991), and the blood cell (Hameed et al. 2018). The specimen was obtained from Qingge fishing port of Qionghai, Hainan province, China (N19°18′48.37″, E110°40′20.21″), and frozen samples and muscle samples were stored in Qionghai research base of Hainan Academy of Ocean and Fisheries Sciences for reference and total DNA extraction.

The whole mitochondrial genome of *A. antiquata* is 45,454 bp in length (GenBank Accession number MK783262) containing 29 tRNA genes, 2 rRNA genes, and 17 protein-coding genes (PCGs). The non-coding region was large and atypical, that is different as expected, with 24,162 bp in length. Except for a tRNA-Asp gene, most of the genes were encoded on the heavy strand. Two overlaps between adjacent genes were found, 4 bp between *COX1* and *ND5*, 50 bp between *ND3* and *ND4L*. The nucleotide base content of *A. antiquata* mitogenome was 27.0% A, 26.7% G, 35.2% T, and 11.1% C. the 62.2% of (A + T) showed great preference to AT.

29 tRNA genes of *A. antiquata* mitogenome vary in size from 55 bp to 73 bp. Seven of them are present more than once: there are 3 copies of *tRNA-Met*, *tRNA-Thr*, *tRNA-Ser*, and *tRNA- Leu* genes, 2 copies of *tRNA-Lys*, *tRNA-Asp*, and *tRNA-Phe*. In addition, a special tRNA gene transfer Pyrrolysine was found in the mitogenome. The 12S rRNA is 631 bp and located between *tRNA-Asn* and *tRNA-Thr*, and the 16S rRNA is 1360 bp, located between *tRNA-Val* and *tRNA-Ala*. Similar to *Anadara cellicata*, among the expected 13 PCGs, 12 were identified, and *ATP8* gene was absent (Kong et al. 2015). In addition, five genes were not annotated with any function. There are five type initiation codons of all PCGs including ATA, ATC, ATT, GTG, and TTG. all PCGs ended with a complete stop codon, 8 use TAA and 9 use TAG. Additionally, 19 microsatellites (SSRs) were identified in *A. antiquata* mitogenome using MISA. Among these SSRs, 12 are mononucleotides (A/T), 4 are dinucleotides (AC/AT/TA), and 3 are trinucleotides (GCT/TCT/TTC). Simultaneously, 9 were detected in the coding region, the others were in the non-coding region.

Using the 12 PCGs encoding by 11 bivalve shellfish, a phylogenetic tree was constructed by maximum-likelihood (ML) method with 1000 bootstrap replicates. The result (Figure 1) showed that the *A. antiquata* was first clustered with *Anadara vellicate*, then with *Tegillarca granosa*. Phylogenetic relationships of the species in Arcidae need more mitogenomes to be clear.

**Figure 1.**
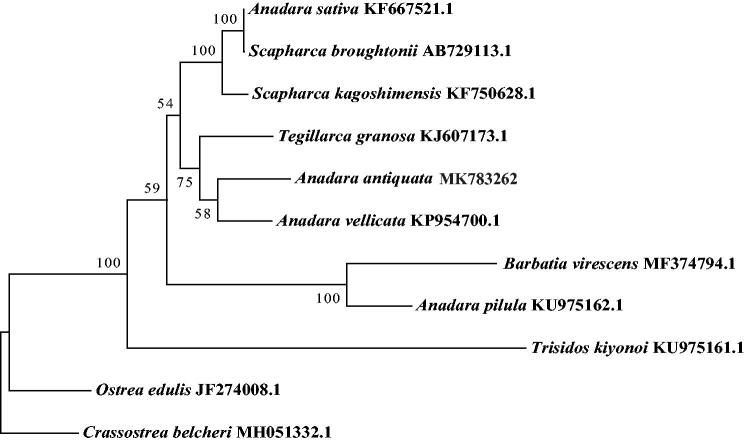
Phylogenetic tree of 11 species based on mitochondrial genome. *Crassostrea belcheri* and *Ostrea edulis* were used as outgroups.
